# Chemsex, G&T, and The Club Drugs Clinic Ireland

**DOI:** 10.1192/j.eurpsy.2022.2077

**Published:** 2022-09-01

**Authors:** K. Santlal, F. Fenton, P. Mccarron, U. Egbuta, E. Keenan

**Affiliations:** 1 St. John of God Hospital, Psychiatry Of Acute & Enduring Illnesses, Dublin, Ireland; 2 HSE National Drug Treatment Centre, Psychiatry, Dublin, Ireland; 3 HSE Primary Care Division, National Social Inclusion Office, Dublin, Ireland

**Keywords:** Gamma Hydroxybutyrate, chemsex, Crystal Methamphetamine, harm reduction

## Abstract

**Introduction:**

Chemsex refers to the intentional consumption of specific substances, Gamma Hydroxybutyrate/Gamma Butyrolactone (GHB/GBL), Crystal Methamphetamine and/or Cocaine to facilitate or enhance the sexual experience. However, there was a plethora of associated problems ranging in severity to complex, life-threatening situations. Since its inception in 2014, The Club Drugs Clinic Ireland, the first outpatient-based clinic for GHB/GBL Detoxification in Ireland, had evolved to include managing problematic chemsex.

**Objectives:**

The Chemsex Working Group Ireland is a collaborative response from governmental and non-governmental agencies. Details of current medical and psychiatric management along with preliminary outcome findings on detoxification, relapse risk and associated factors will be presented.

**Methods:**

Data collected include socio-demographic variables, gender and sexuality, detoxification setting, relapse history and attendance for counselling or aftercare. Descriptive analyses were conducted on referral counts, drug trends, success of first treatment episode, subsequent relapse rate, and uptake of counselling and aftercare.

**Results:**

There have been over 200 referrals to the Club Drugs Clinic Ireland. A number of predisposing and precipitating factors, contributed to the relapse rate (up to 70%) both in Ireland and internationally. The salutogenic, biopsychosocial-based model of addiction recovery produced the best outcomes. This integrated Dual Diagnosis Psychiatry, Sexual Health Medicine, Emergency Medicine and external services for a more comprehensive care.

**Conclusions:**

The pattern of referrals reflects population trends in chemsex, despite the COVID-19 restrictions. While detoxification is largely successful, the high relapse rate highlights the challenge of maintaining abstinence. In order to competently address problematic chemsex, service coordination across various medical professions and ongoing monitoring of the substances consumed is quintessential.

**Disclosure:**

No significant relationships.

